# Indigo Carmine in a Food Dye: Spectroscopic Characterization and Determining Its Micro-Concentration through the Clock Reaction

**DOI:** 10.3390/molecules27154853

**Published:** 2022-07-29

**Authors:** Maja C. Pagnacco, Jelena P. Maksimović, Nenad T. Nikolić, Danica V. Bajuk Bogdanović, Milan M. Kragović, Marija D. Stojmenović, Stevan N. Blagojević, Jelena V. Senćanski

**Affiliations:** 1 Institute of Chemistry, Technology and Metallurgy, University of Belgrade, Njegoševa 12, 11000 Belgrade, Serbia; 2 Faculty of Physical Chemistry, University of Belgrade, Studentski Trg 12-15, 11000 Belgrade, Serbia; jelena.maksimovic@ffh.bg.ac.rs (J.P.M.); danabb@ffh.bg.ac.rs (D.V.B.B.); 3 Institute for Multidisciplinary Research, University of Belgrade, Kneza Višeslava 1, 11030 Belgrade, Serbia; nnikolic@imsi.bg.ac.rs; 4 “Vinča“ Institute of Nuclear Sciences, National Institute of the Republic of Serbia, University of Belgrade, Mike Petrovica Alasa 12-14, 11351 Belgrade, Serbia; m.kragovic@vinca.rs (M.M.K.); mpusevac@vinca.rs (M.D.S.); 5 Institute of General and Physical Chemistry, University of Belgrade, Studentski Trg 12-15, 11000 Belgrade, Serbia; sblagojevic@iofh.bg.ac.rs

**Keywords:** blue dye, indigo carmine, Raman spectroscopy, geometrical optimization, UV/Vis, Briggs–Rauscher reaction

## Abstract

Indigo carmine is a commonly used industrial blue dye. To determine its concentration in a commercially available food dye composed of a mixture of indigo carmine and D-glucose, this paper characterizes it through (ATR, KBr) FTIR micro-Raman as well as UV/Vis and clock: Briggs–Rauscher (BR) oscillatory reaction methods. The indigo carmine was detected in the bulk food dye only by applying micro-Raman spectroscopy, indicating a low percentage of the indigo carmine present. This research provides an improvement in the deviations from the experimental Raman spectrum as calculated by the B97D/cc-pVTZ level of theory one, resulting in a better geometrical optimization of the indigo carmine molecule compared to data within the literature. The analytical curves used to determine indigo carmine concentrations (and quantities) in an aqueous solution of food dye were applied by means of UV/Vis and BR methods. BR yielded significantly better analytical parameters: 100 times lower LOD and LOQ compared to commonly used UV/Vis. The remarkable sensitivity of the BR reaction towards indigo carmine suggests that not only does indigo carmine react in an oscillatory reaction but also its decomposition products, meaning that the multiple oxidation reactions have an important role in the BR’s indigo carmine mechanism. The novelty of this research is the investigation of indigo carmine using a clock BR reaction, opening new possibilities to determine indigo carmine in other complex samples (pharmaceutical, food, etc.).

## 1. Introduction

To make products more attractive for consumers, synthetic dyes are added. Products that are in color attract customer attention more than those which are not; this is especially the case for products aimed at children. These dyes are particularly present in processed foodstuffs (candy, desserts, beverages, etc.) as well as in pharmaceutical goods (pills and tablets) [1,2].

Organic coloring agents in the pharmaceutical and food industry are assigned the letter E. These may also include food additives such as vitamin C and are listed under “ingredients” on the product’s packaging [3]. The European Free Trade Association is tasked with evaluating the safety of all ingredients and preservatives within perishable items sold in the European common market [4].

Blue dye is noted as E131, 132, and 133, and there is a subtle difference between these three; the E132 is more toxic than the others listed [5]. Apart from the use of E132 (indigo carmine, indigotine) as a blue dye for the above-mentioned samples, there is a medical use for this dye as well [6,7,8]. The safe indigo carmine dose for human consumption is considered to be 500 mg/kg [7]. As a dye commonly used in the textile industry [9], it could be found in wastewater [10].

**Figure 1:**
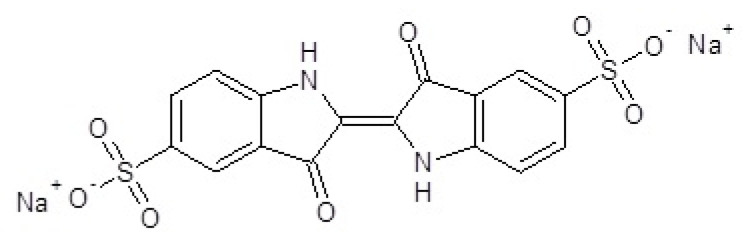
Indigo carmine molecule ((2E)-3-oxo-2-(3-oxo-5-sulfonato−1,3-dihydro-2H-indol-2-ylidène)-5-indolinesulfonate de disodium.

The chemical formula of indigo carmine [11] ((2E)-3-oxo-2-(3-oxo-5-sulfonato-1,3-dihydro-2H-indol-2-ylidène)-5-indolinesulfonate de disodium), possessing a molar mass of 466.36 g/mol, is presented in Figure 1 [12].

While this dye was once exclusively extracted from the Isatis plant [13], it is now synthetically produced. Regarding the common usage of this color in many fields, the authors want to deeper investigate (using different techniques and methods) the indigo carmine in a food dye sample.

Due to the potential toxicity and harmful effects of indigo carmine on the ecosystem and biodiversity, its presence and concentration must be reliably determined. Different methods have been proposed to identify and quantify indigo carmine in wide-range samples, based mainly on UV/Vis spectrometry [14,15,16,17,18], electrochemical techniques [19,20,21], chromatography [22,23,24], FTIR spectroscopy [12,25], as well as advanced digital imaging techniques [26,27]. Understandably, all of these techniques have different sensitivity and accuracy of indigo carmine detection mainly depending on sample nature (to be more precise on its matrix). Many of the techniques listed are expensive or demand complex sample treatment. Therefore, there is always a demand for new techniques that would be successful in identification and quantification, while being affordable and low-cost. The paper presented aims to introduce the Briggs–Rauscher clock reaction as a possible technique for indigo carmine quantification. This is pioneering work in food dye examination, because to the best of the authors’ knowledge, a clock reaction has not been used to determine the concentration of any dye. 

A chemical clock reaction is a process where a periodic change in intermediate concentration (or specific color) happens with the progress of a reaction [28]. One of the methods here applied is the clock Briggs–Rauscher (BR) oscillatory reaction method [29]. It is a well-known method for determining unknown concentrations of an active (reactive) analyte [29,30,31], its antioxidant/antiradical activity [32,33,34,35,36,37], as well as distinguishing insoluble material such as bronzes [38,39,40] and bentonite clays [41]. However, this is the first attempt to use the BR reaction for determining some dyes (in a commercial sample). The presented investigation is more interesting because it was performed “backwards”, meaning that we do not know the exact quantities of the commercially available food dye. Due to the package of food dye containing a mixture of indigo carmine and D-glucose in an unknown amount, the aim of this work was to determine the unknown concentration of indigo carmine. Therefore, this paper deals with the investigation of a food dye sample by using different techniques (infra-red (IR) and Raman spectroscopy, ultraviolet (UV)-visible (VIS) as well as the BR reaction method). The other important outcome comes from applying density functional theory (DFT) calculation of a Raman spectrum and better geometrical optimization of indigo carmine, achievement in this work compared to the reference [42].

It is expected that this study could provide a reference for the spectral analysis of indigo carmine in a commercially available food dye sample and pave the way for determining indigo carmine in the Briggs–Rauscher oscillatory reaction. 

## 2. Materials and Methods

The experimental part included tasks given in Figure S1 in Supplementary Material describing the characterization of a commercial food dye sample and establishing the analytical curves to determine the micro-concentrations of indigo carmine

The materials used in this experiment were standard indigo carmine (Alfa Aesar A16052), D-glucose (Lučar, Novi Sad, Serbia), and food dye (mixture of indigo carmine and D-glucose, producer: MIP Market, Belgrade, Serbia).

### 2.1. Infra-Red (IR) Spectroscopy 

#### 2.1.1. Attenuated Total Reflection (ATR)-Fourier-Transform Infrared (FTIR) Spectroscopy

An ATR FTIR measurement was carried out using an iS20 Thermo Nicolet (using a diamond crystal at 32 scans per sample and a 2 cm^−1^ resolution in the range of 400 to 4000 cm^−1^). 

#### 2.1.2. FTIR Spectroscopy-KBr Pellet

A recording of the indigo carmine using KBr FTIR was carried out under AVATAR 370 FTIR equipment. A KBr lozenge was made using 1 mg of the sample mixed with 150 mg of the KBr. The sample’s spectra were collected in the range of 4000–400 cm^−1^ at 64 scans and a spectral resolution of 2 cm^−1^.

### 2.2. Raman Spectroscopy

#### 2.2.1. Micro Raman Spectroscopy

The spectra were recorded using a Thermo Scientific DXR Raman microscope, performed at λ = 532 nm, a laser strength of 8 mW, 10× magnification 1, and a 2.1 µm laser spot in the range of 400–1800 cm^−1^. A spectrograph under 900 lines mm^−1^ grating analyzed the scattered light. The spectra were recorded under 10 s exposure time and 10 exposures per spectrum. 

#### 2.2.2. Raman-Spectrum Optimization and Calculation

The indigo carmine’s geometry optimization was performed first to achieve an optimal geometry of the compound under the B97D/cc-pVTZ level of theory. The calculations were carried out in Gaussian 09, Revision D.01 [43]. Following their optimization, the Raman spectrum was also calculated under the B97D/cc-pVTZ level of theory. 

### 2.3. UV-Vis Measurements

All UV/Vis measurements were obtained through ultraviolet-visible (UV/Vis) spectroscopy using an Agilent 8453 spectrophotometer containing a diode-array detector at room temperature (~25 °C). The range used was 200–1100 nm. 

#### 2.3.1. The Standard Calibration Curve of Indigo Carmine

The standard chemical of indigo carmine was Alfa Aesar A16052. Firstly, 0.0035 g of indigo carmine was dissolved in bi-distilled water in a 250 mL flask. The concentrations used to obtain an analytical curve were 3.00 × 10^−5^ mol dm^−3^; 1.80 × 10^−5^ mol dm^−3^; 1.50 × 10^−5^ mol dm^−3^ and 6.00 × 10^−6^ mol dm^−3^.

#### 2.3.2. The D-Glucose UV/Vis Spectrum 

Then, 0.4895 g of pure D-glucose (dextrose) (Lučar, Serbia) was dissolved in 25 mL of bi-distilled water, whereby the UV/Vis spectrum was recorded.

#### 2.3.3. The Food-Dye UV/Vis Spectrum 

To obtain a blue-colored solution, 0.0464 g of commercially available food dye (producer, MIP Market, Serbia) composed of a mixture of indigo carmine and D-glucose in an unknown percent was dissolved in 25 mL bi-distilled water. To determine the amount of indigo carmine by the UV/Vis method in the food dye, the primary solution (0.0464 g of the food dye in 25 mL) was diluted tenfold in order to fit within an analytical curve.

### 2.4. The Oscillatory Briggs-Rauscher Reaction and Experimental Conditions

The dynamic behavior of the BR reaction was followed potentiometrically, using a Pt electrode (METHROM AG. Serial No. 6.0301.100, Switzerland) and a double junction Ag/AgCl electrode (METHROM AG. Serial No. 6.0726.100, Switzerland) as a reference in a closed, well-stirred (900 rpm) system and thermostated at 37.0 °C. 

All reagents used to prepare the solutions were of analytical grade. Initial concentrations of reactants were [C_3_H_4_O_4_]_0_ = 0.0789 mol/dm^3^, [MnSO_4_]_0_ = 0.0075 mol/dm^3^, [HClO_4_]_0_ = 0.0300 mol/dm^3^, [KIO_3_]_0_ = 0.0752 mol/dm^3^, and [H_2_O_2_]_0_ = 1.176 mol/dm^3^. The reaction volume was 25 mL. The substances were added into the reaction vessel in the following order: C_3_H_4_O_4_, MnSO_4_, HClO_4_, and KIO_3_. Hydrogen peroxide was added to the reaction vessel only after both the temperature and the Pt potential were stabilized, which was taken to be the start of the BR reaction. In order to investigate the influence of the food dye (the mixture of indigo carmine and D-glucose) on the BR oscillatory dynamics, two experimental types were carried out for 30 s after the addition of the hydrogen peroxide:

- Pure D-glucose (100 μL of 0.1 mol dm^−3^ aqueous solution) was injected;
- the food dye dissolved in bi-distilled water indigo carmine concentrations in BR solution from 2.62 × 10^−7^ mol dm^−3^ to 1.04 × 10^−6^ mol dm^−3^ was examined.

## 3. Results and Discussion

In order to determine the concentration of indigo carmine present in the food dye, an analysis was performed using a ranged spectrum of robust to sensitive techniques. 

### 3.1. ATR FTIR Spectroscopy and KBr FTIR Technique 

As an organic compound that possesses functional groups, indigo carmine is observable in the infrared (IR) spectrum. Hence, ATR FTIR spectroscopy was used as the most robust technique to detect and characterize indigo carmine [44,45]. Due to the presence of indigo carmine in the food dye mixed with D-glucose, both spectra of the food dye and that of the D-glucose are presented in Figure S2a. These spectra are also compared to the ATR FTIR reference spectrum of D-glucose from the database [46] as well as for the pure indigo carmine [47]. According to Figure S2a, the ATR FTIR shows the D-glucose overlapping across the spectrum of the food dye, thereby indicating the presence of a limited concentration of indigo carmine in the food dye sample. KBr as a technique was also carried out to achieve a higher sensitivity as the entirety of the radiation goes through the sample. As presented in Figure S2b, the KBr technique yielded an overlapping spectrum as well, suggesting the presence of a minor concentration of indigo carmine. Therefore, FTIR is able to detect indigo carmine but not its concentration, thereby making it of limited use in the investigation. All the peaks of dextrose obtained for both techniques have been assigned, compared, and presented in Table S1 in the Supplementary Material.

### 3.2. Raman Spectroscopy

**Figure 2:**
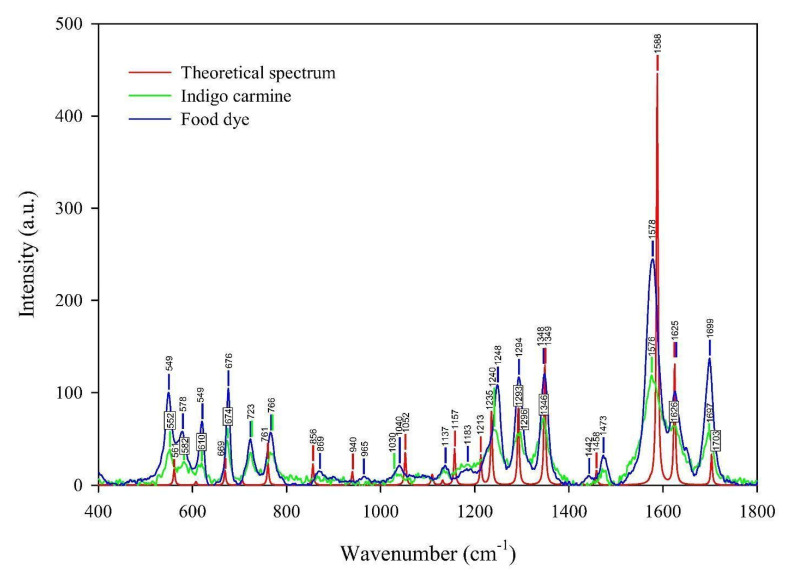
The experimental Raman spectra of the indigo carmine standard and the food dye indigo carmine, together with the theoretical calculated Raman spectrum of standard indigo carmine.

**Figure 3:**
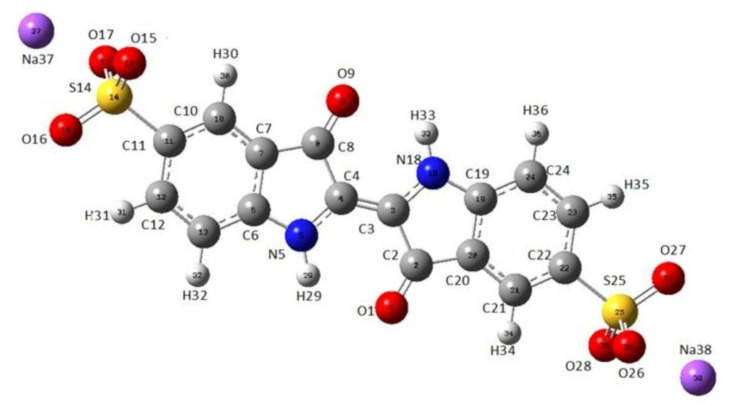
The optimized geometry of indigo carmine using B97D/cc-pVTZ level of theory.

*As a technique that may “select” the pure component in a mixture*, the micro-Raman technique was applied to characterize indigo carmine. To confirm that pure indigo carmine was selected and the Raman spectrum of indigo carmine was obtained, the Raman spectrum of a standard indigo carmine sample was recorded. Therefore, the food dye spectrum recorded was compared to the standard indigo carmine Raman spectrum (Figure 2). DFT calculation of the indigo carmine Raman spectrum was then carried out. In order to calculate the spectrum, a geometry optimization was first performed (Figure 3), and the bounds and angles of the molecule optimized are listed in Table S2 in the Supplementary Material. The assignment of the Raman peaks of the indigo carmine was performed in order to characterize the compound investigated (Table 1). The theoretical spectrum of indigo carmine is presented in Figure 2, together with indigo carmine and the spectrum of the food dye recorded by micro-Raman spectroscopy.

The molecular lengths and bonds of indigo carmine are listed in Table S2 of the Supplementary Material. However, it is challenging to present a true geometry optimization in terms of bond lengths and angles between atoms when a global energy minimum is achieved.

Based on the peak positions of the indigo carmine spectrum calculated compared to that of the experimental, the band positions indicate good agreement (Figure 2). The bands of the calculated spectrum as well as the data found in the literature [42] ranging from 1000 to 2000 cm^−1^ are listed in Table S3. Table S3 lists the calculated and experimental spectra which show good agreement. The bands that have a lower or the same deviation compared to the data found in the literature are 1348, 1588, 1625, and 1703 cm^−1^ (also high intensities). A lower deviation compared to the literature’s data was anticipated due to the higher basis set applied in the calculation of the indigo carmine spectrum used in this study compared to that used in the reference [42]. Owing to the lower deviations obtained in our study, the authors assigned the bands of the theoretical Raman spectrum of indigo carmine in Facio 2313–64 software (Table 1).

The lower distinction between the vibration peaks from the spectrum calculated and the experimental compared to the data found in the literature indicates a better geometry optimization was achieved.

According to the results obtained from the Facio software, there are multiple complex vibrations overlapping for each band. Namely, the singular band corresponds to more than one vibration of molecule groups, as listed in Table 1, column 4. It is important to note that not all the vibrations of the functional groups presented in the fourth column appear in the literature cited. Moreover, the multimedia materials illustrating the complex molecule vibrations (video1.mp4–video4.mp4) for the most intense peaks are given in the Supplementary Material (Figure S3).

### 3.3. UV/Vis and Briggs-Rauscher Methods

When the presence of indigo carmine was confirmed using a technique that may detect micro-concentrations, the construction of an analytical curve was followed to determine the micro-concentration of the indigo carmine. The measurements were performed by UV/Vis, a technique often used to detect micro-concentrations in samples. The results obtained make this analytical curve of standard indigo carmine acceptable for measuring micro-concentrations of indigo carmine (*A* = 9000 mol^−1^ dm^3^ × *C* + 0.006, where *A* is absorbance and *C* is the concentration of indigo carmine standard dissolved in water, Figure S4). Taking into account formula A = abc (where b is an optical path of 1 cm), the molar absorption coefficient for indigo carmine dissolved in water, a = 9 × 10^3^ mol^−1^ dm^3^ cm^−1^, was obtained through an analytical curve slope, whose value is in good agreement with the reference [57] (8.1 × 10^3^ mol^−1^ dm^3^ cm^−1^). In order to determine the unknown concentration of indigo carmine in the food dye (i.e., a mixture of dextrose and indigo carmine), a solution of the food dye sample was recorded using UV/Vis. It was found that the absorbance of the indigo carmine was A = 0.12 at 611 cm^−1^ for a solution of 0.00464 g of the food dye in 25 mL of the aqueous solution. The calculated unknown percentage of indigo carmine in the food dye sample was found to be 3.21%.

To prove this method applicable in determining the indigo carmine present in the food dye, a UV/Vis spectrum of pure D-glucose was recorded as well as a spectrum of the food dye (Figure S5 in Supplementary Material). Due to the former spectrum not affecting the latter (i.e., D-glucose contains no chromospheres that react with the UV/Vis region), this method may be used to determine indigo carmine in this particular food dye. Based on the results obtained, a calibration curve was recorded from the food dye dissolved in water. The parameters of the UV/Vis analytical curve as well as its LOD and LOQ are laid out in Table 2, where indigo carmine food dye was used to calibrate the analytical curve. The LOD value is the lowest concentration of the analyte detectable at a specified level of confidence. LOQ is the limit of the lowest concentration determinable but maintains an acceptable level of repeatable precision and trueness [58].

Following the UV/Vis determining the presence of indigo carmine, the experiment was carried out using the Briggs–Rauscher reaction, as a method that may also determine micro-concentrations in samples. To the best of the authors’ knowledge, this study is the first to have determined the presence of indigo carmine using the BR reaction.

**Figure 4:**
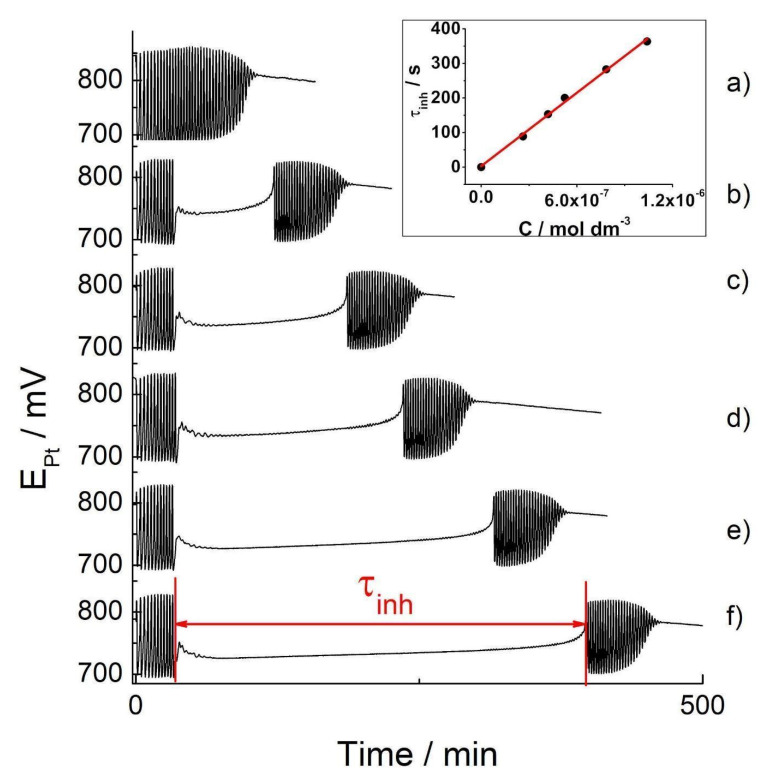
The Pt Electrode BR oscillograms: the basic BR oscillogram (**a**) and the oscillograms obtained by the addition of tested concentrations of the indigo carmine in food dye: 2.62 × 10^−7^ mol dm^−3^ (**b**); 4.19 × 10^−7^ mol dm^−3^ (**c**); 5.24 × 10^−7^ mol dm^−3^ (**d**); 7.84 × 10^−7^ mol dm^−3^ (**e**); 1.04 × 10^−6^ mol dm^−3^ (**f**): inserted picture: the calibration curves obtained from the concentrations of indigo carmine of the food dye when added to the oscillating Briggs–Rauscher reaction.

The recorded Pt-potential vs. time series (or oscillogram) of the BR reaction when the analyte is not added (i.e., the basic oscillogram) was performed threefold (see Supplementary Material S6a). The oscillatory reaction, under applied experimental conditions (see Materials and Methods), shows good reproducibility (Figure S6a). When hydrogen peroxide is added, the BR system immediately oscillates. After approximately 107 s, the oscillatory dynamics finish. Given that the Briggs–Rauscher oscillatory reaction (and oscillatory reactions in general) is extremely sensitive to the presence of external substances (perturbations), which means not high selectivity, it is important to examine the influence of other substances (interferents, matrix) that may be present in samples that are analyzed by the technique proposed. Due to the presently investigated food dye sample consisting of only two ingredients: D-glucose (dextrose) and indigo carmine, we investigated the influences of D-glucose on the BR oscillatory response. The effects of the added D-glucose in the basic BR oscillogram are shown in Figure S6b. The addition of D-glucose (100 µL, 0.1 mol/dm^3^) at the 30 s mark in the BR reaction bears no effect on the reaction itself (Figure S6b). The number of oscillations (39), as well as the oscillatory time (107 s), remains unchanged when D-glucose is added to the BR system. Hence the D-glucose has no effect on the BR system; an aqueous solution of the food dye may be used to obtain an analytical curve of indigo carmine. The effect of the range of indigo carmine concentrations in food dye on the oscillatory BR reaction is presented in Figure 4.

According to the oscillograms obtained, the addition of the dye at the 30 s mark of the BR reaction significantly changes its dynamics (Figure 4b–f). Namely, it immediately quenched the oscillatory behavior but only for a determinant time, after which the oscillations occurred once more. However, the secondary oscillations showed different amplitudes and time periods between them. As marked in Figure 4, the time for which the oscillating regime is temporarily quenched is the inhibitory period, τ_inh_. Its appearance may be successfully utilized to calibrate curve making. The inhibitory period (τ_inh_) as a linear function of the concentrations of the dye is presented in Figure 4 inset, while the curve parameters are presented in Table 2. Since the D-glucose does not affect the BR reaction, the obtained linear dependence, τ_inh_ = (4.0 ± 0.1) × 10^8^ × C_ind_ + (3 ± 6) may be utilized to determine any unknown concentrations of indigo carmine in a range of commercial blue food dye samples containing indigo carmine. The characteristic parameters of the BR analytical curve, together with LOD and LOQ values, are given in Table 2.

The results obtained for the LOD and LOQ from both the UV/Vis and BR method (see Table 2) indicate significantly better characteristics for the BR technique: 100 times lower LOD and LOQ compared to the UV/Vis technique. These results make the BR reaction more acceptable when determining micro-concentrations of indigo carmine compared to the UV/Vis method that is commonly used. Therefore, the BR reaction could be satisfactorily used for the quantification of indigo carmine in a commercial food dye sample (for cake-decorating purposes), which is a mixture of D-glucose and indigo carmine. The abilities of the BR method to determine indigo carmine concentrations in complex food or pharmaceutical samples should be addressed in future work.

### 3.4. The Degradation of Indigo Carmine in a Briggs-Rauscher Oscillatory Reaction and Its Remarkable Sensitivity

**Figure 5:**
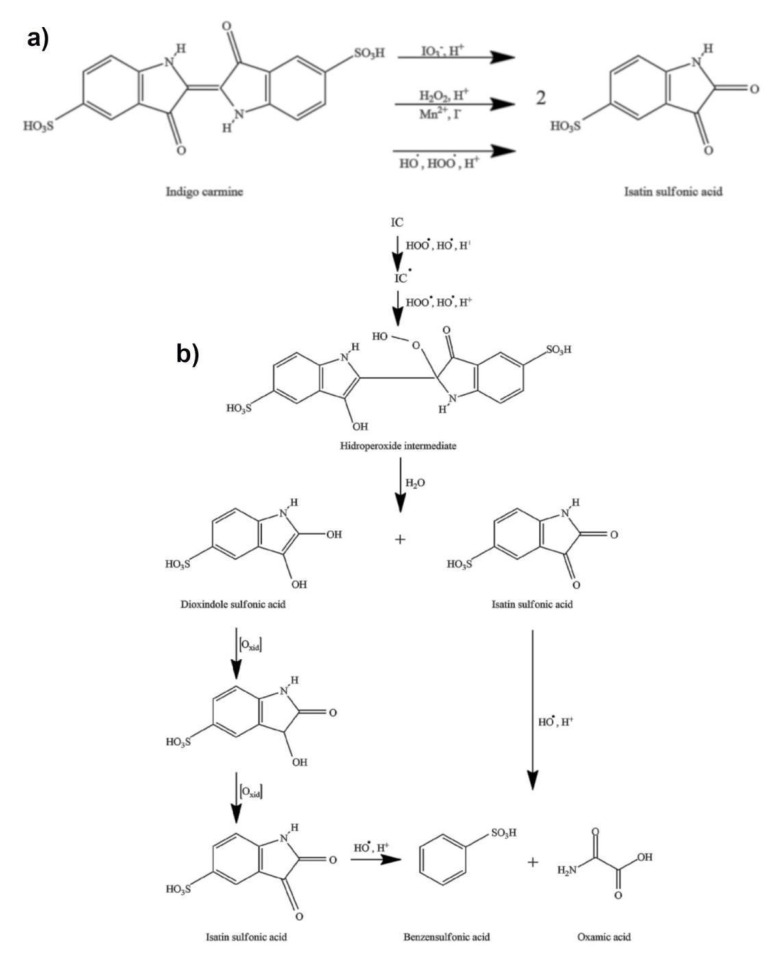
(**a**) the possible reaction pathways of indigo carmine oxidation in a Briggs–Rauscher oscillatory reaction (**b**) the mechanism of indigo carmine (and its derivative) action with HOO• /HO• radicals. in the Briggs–Rauscher (BR) reaction (for more details see references [65,66,67,68]). The multiple oxidation reactions account for the high sensitivity of the BR towards indigo carmine.

Due to the presence of iodine in a + 5 oxidation state (iodate), as well as the reactive radical species (HO•, HOO•, IO_2_•), the Briggs–Rauscher oscillatory reaction is a highly oxidative medium [59,60,61,62,63,64]. Therefore, the addition of indigo carmine into the BR oscillatory reaction unambiguously leads to its oxidation. There are three possible pathways of indigo carmine oxidation in an acidic BR solution supported by the literature (Figure 5a). Indigo carmine may be oxidized with: (i) iodate [65], (ii) hydrogen peroxide [66], and/or (iii) a reactive oxygen species (HOO•, HO•) [67]. As presented in Figure 5a, all reactions bear the same final product of isatin sulfonic acid (ISA).

Any indigo carmine concentration that disturbs the BR oscillogram is less than 10^−5^ M. Further, a concentration of indigo carmine ≤10^−6^ M may cease the oscillatory behavior for ~400 s, which is considerable in its duration as the entire BR reaction occurs over 106 ± 5 s (see basic oscillogram, Figure 4a). Consequently, the inhibitory period persists until all indigo carmine is consumed, making it only partially accurate in terms of its kinetics. This begs the question: “How is it possible that the concentration of 10^−6^ M is able to maintain an inhibition for 400 s?”. This phenomenon indicates that indigo carmine is not solely responsible for the behavior obtained and that its oxidation/degradation products play an important role in the inhibition of the BR oscillatory reaction.

An accurate mechanism of the indigo carmine reaction with an iodate in an acidic solution is unknown. Generally, a mechanism implies there to be a two-electron loss from the indigo carmine to the isatin sulfonic acid, while the iodate (IO_3_^−^) could be reduced to iodous/iodite (IO_2_^−^) ions [68]. The order of the iodate-indigo carmine reaction in an acidic solution (such as a BR system) was found to be four: one order each with respect to the indigo carmine and the iodate ion as well as the second order with respect to the H^+^ ion. These indicate a complex reaction mechanism. Additionally, the iodate-indigo carmine reaction rate constant is found to be 3.49 × 10^−2^ dm^3^ mol^−3^ s^−1^ [65]. Taking into account the rate constant observed and the iodate concentration in the BR system (7.5 × 10^−2^ M), as well as the low concentration of indigo carmine (<<10^−5^ M) affecting the oscillatory dynamics, the iodate-indigo carmine reaction (although existing in the BR system) is not the dominant reason for the inhibition observed. This conclusion may be made based on the low rate of the overall iodate-indigo carmine reaction.

Likely due to the high concentration of hydrogen peroxide (1.27 M), as well as the presence of iodide (I-) and manganese (Mn2+) ions in a BR system, the two remaining pathways (indigo carmine with hydrogen peroxide and indigo carmine with HO•, HOO•) are more likely to occur in a BR system. These ions strongly catalyze the decomposition of hydrogen peroxide through the production of HO• and HOO• radicals [59,60,61]. Therefore, the last two pathways may be considered to be only one (Figure 5b). What is more, every HO• formed (from H_2_O_2_) would quickly be converted to HOO•: HO• + H_2_O_2_ → HOO• + H_2_O, *k = 4.5 × 10^7^* *M^−1^ s^−1^* [60]. Thus, the HOO• radical may be considered to be a dominant intermediate (in relation to H_2_O_2_ and HO•) reacting with the indigo carmine and its derivative. Finally, the oxidation of the indigo carmine with the hydrogen peroxide and/or the HOO• and HO• is almost certainly transformed into a hydroperoxide intermediate in the acidic solution as presented in Figure 5b.

The formation of a hydroperoxide intermediate involves a simultaneous electrophilic attack of hydroxyl and hydroperoxyl radicals (both highly present in the BR) from the indigo carmine double carbon–carbon bond [67]. This key intermediate could further hydrolyze to isatin sulfonic acid and dioxindole sulfonate. The dioxindole sulfonate could oxidize to isatin sulfonic acid (as outlined in Figure 5b). Therefore, there are at least “six oxidation processes” for one molecule of indigo carmine, which involves the oxidation of dioxindole sulfonate to isatin sulfonic acid and opening the nitrogen ring in the isatin sulfonic acid reaction with the hydroxyl radical (see Figure 5b). Hence, the addition of indigo carmine in the BR reaction does not only reflect the concentration of indigo carmine but all molecules may be produced themselves from the indigo carmine. This conclusion is important for the oscillatory dynamics, as it is the same reason why there is a low (<<10^−5^ M) concentration of the indigo carmine affecting the oscillatory dynamics. Consequently, the multiple oxidation reactions may account for the remarkable sensitivity of the BR method towards indigo carmine.

## 4. Conclusions

This work aims to determine the micro-concentration of indigo carmine in food dye (a mixture of indigo carmine and D-glucose in unknown amounts) through ATR FTIR and KBr FTIR, micro-Raman spectroscopy, UV/Vis, and the clock reaction Briggs–Rauscher kinetic method. Additionally, the better optimization and geometry of the indigo carmine molecule compared to data found in literature was achieved by the B97D/cc-pVTZ level of theory, thereby improving the data obtained for the vibrational bands. The UV/Vis method found the indigo carmine quantity to be 3.21% in a commercially available sample of food dye. Indigo carmine has not been investigated by the Briggs–Rauscher oscillatory reaction, making this paper novel in its research. Comparing the results obtained from the UV/Vis and BR methods, there are significantly better characteristics for the BR method; specifically, a 100-fold lower LOD and LOQ related to the UV/Vis technique. The Briggs–Rauscher oscillatory reaction is, therefore, more acceptable to determine micro-concentrations of indigo carmine. This remarkable sensitivity of the BR reaction towards indigo carmine suggests that not only may indigo carmine be detected in oscillatory reaction, but also its decomposition products.

## Figures and Tables

**Table 1:** Assignation of vibrations from the Raman spectrum of food dye indigo carmine, standard indigo carmine, and the calculated Raman spectrum for and vibrations found in Facio and comparison with literature data.

Food Dye	INDIGO Carmine Exp.	Spectrum Calculated	Band Vibrations from Facio Obtained in This Work	Ref.
Spectrum	Spectrum			
Raman shift [1/cm]	Raman shift [1/cm]			
549	552	561	C-C ring bending: weak; C-H bending: weak; N-H bending: weak; S-O bending: weak	[42,48,49]
676	674	669	C-C ring bending: weak; C-H bending: weak; N-H bending: weak	[31,48,49]
723	723	705	N-H bending: medium; C-H bending: medium; S-C stretching: weak	[49]
766	766	761	C-C ring stretching: weak; C-H bending: weak	[42,48,49,50,51,52,53]
869	864	856	C-C-C ring bending: weak; C-N bending: weak	[42,49]
1040	1030	1052	C-H bending: strong; C-S stretching: medium; C-C ring stretching: weak	[42,48,49]
1137	1137	1157	C-H bending: strong; S-O stretching: weak	[48,49,53]
1183	1183	1157	C-C ring stretching: medium; C-H bending: strong; N-H bending: weak	[42,48,49,50,51,52]
1248	1240	1235	C-H bending: strong; N-C asymmetrical stretching: medium; C-C ring stretching: weak	[42,48,49,50,51,52,53]
1294	1296	1293	C-H bending; C-C ring stretching; N-H stretching	[42,48,49,51,53]
1348	1346	1349	C3 = C4 stretching: strong; C-C ring stretching; C-N ring stretching; C-H bending	[42,48,51,52]
1442	1473	1458	C-C ring stretching; C-H bending: strong C13-H32, strong C24-H36; N-H bending: weak N5-H29, weak N18-H33	[42,48,49,50,52,53,54]
1578	1576	1588	C = C ring-ring stretching: strong C3-C4; C-C ring stretching; C = O stretching; N-H bending	[42,48,49,50,51,52,53,54,55,56]
1625	1626	1625	C = C ring-ring stretching: strong C3-C4; C-C ring stretching; C = O ring symmetrical stretching	[42,48,49,50,51,52,53]
1699	1697	1703	C = C ring-ring stretching: strong C3-C4; C = O ring symmetrical stretching: strong C2-O1, strong C8-O9	[42,48,49,50,51,52,53,54,55]

**Table 2:** A comparison of the two different methods used to determine indigo carmine concentration in the food dye sample.

UV/Vis Spectrophotometric Method				
A = n + kC	n	Error n (Sn)	k(slope) (Mol^−1^ dm^3^)	Error k (Sk) (Mol^−1^ dm^3^)
*A = 1.000 + 9000* × *C*	1.000	0.005	9000	100
	LOD = 3.3 × Sn/k		LOQ = 10 × Sn/k	
	1.8 × 10^−6^ mol dm^−3^		5.7 × 10^−6^ mol dm^−3^	
Briggs–Rauscher oscillatory method				
τ_inh_ = n + kC	n(s)	error n (Sn) (s)	k(slope) (mol^−1^ dm^3^ s)	error k (Sk) (mol^−1^ dm^3^ s)
τ_inh_ = 3 + 4 × 10^8^ × C_ind_	3	2	4 × 10^8^	1 × 10^7^
	LOD = 3.3 × Sn/k		LOQ = 10 × Sn/k	
	1.65 × 10^−8^ mol dm^−3^		5 × 10^−8^ mol dm^−3^	

## Data Availability

Not applicable.

## Supplementary Materials

The following supporting information can be downloaded at: https://www.mdpi.com/article/10.3390/molecules27154853/s1. References [69,70] are cited in the supplementary materials.
