# Supplementary material for: Indigo Carmine in a Food Dye: Spectroscopic Characterization and Determining Its Micro-Concentration through the Clock Reaction
Source: Molecules. 2022 Jul 29;27(15):4853. doi: 10.3390/molecules27154853 (PMC9369777; doi:10.3390/molecules27154853)
Supplement: Supplementary file 1 [file molecules-27-04853-s001.zip › molecules-1804332-supplementary-resubmit.pdf]

SUPPLEMENTARY MATERIAL

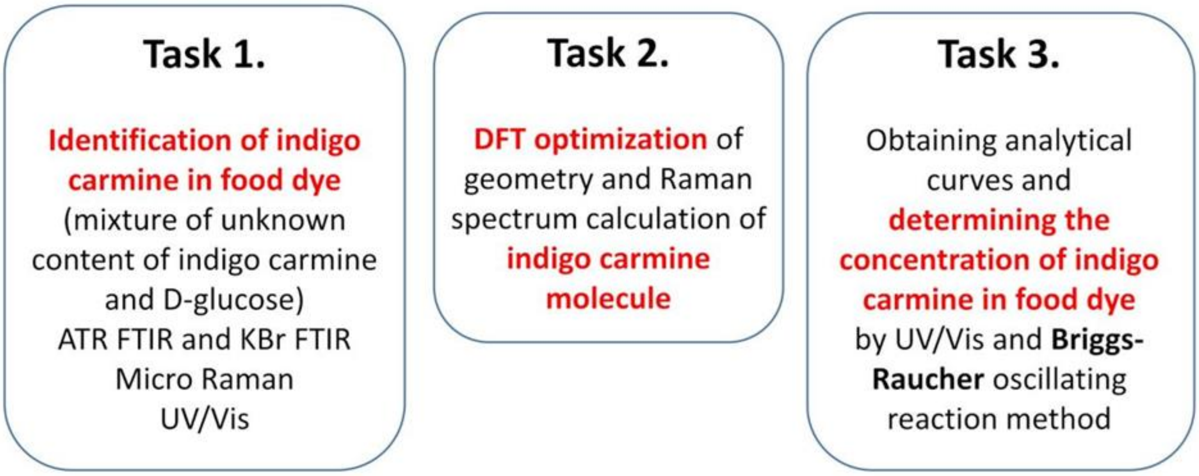

Figure S1. The flow of experimental tasks .

ATR AND KBr FTIR SPECTROSCOPY

ATR was done first as it is the most robust technique Since it did not yield conclusive results, KBr FTIR, a more sensitive technique, also did not detect indigo carmine. These preliminary results suggested that indigo carmine is present in low concentrations in the food dye. The results obtained for the ATR and KBr measurements of the food dye are presented in Figure S2. a) and Figure S2 b). T he vibrations of molecular groups are listed in Table S1.

THE PEAKS OF THE INFRARED SPECTRUM OF D-GLUCOSE (DEXTROSE)

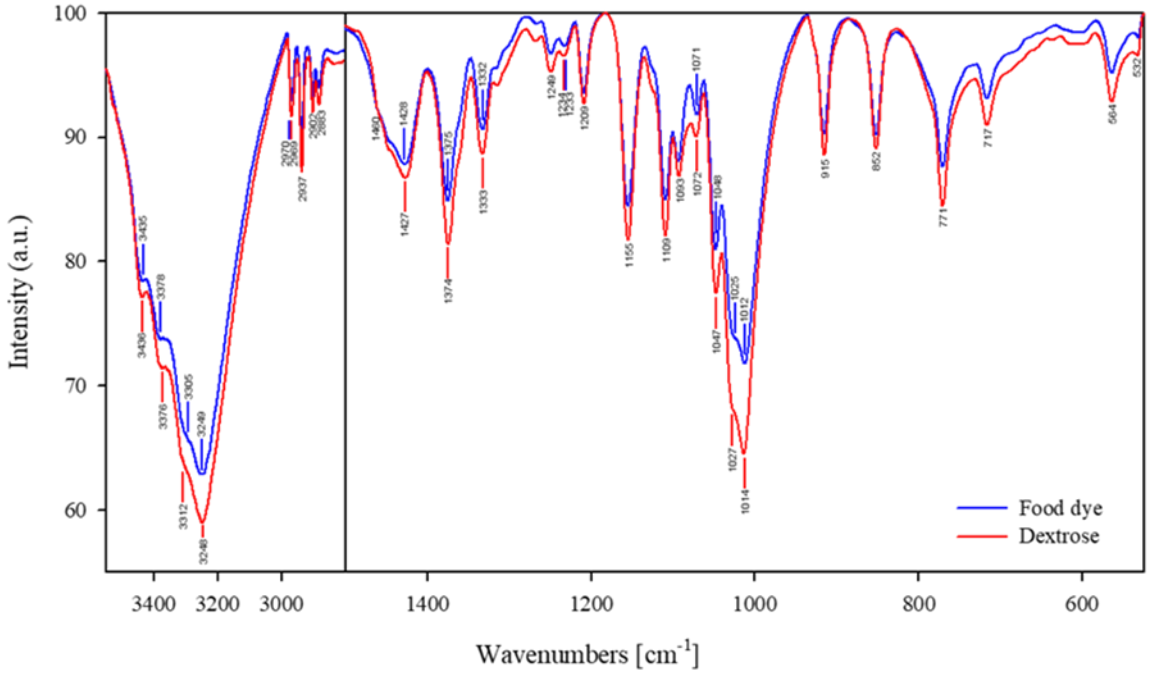

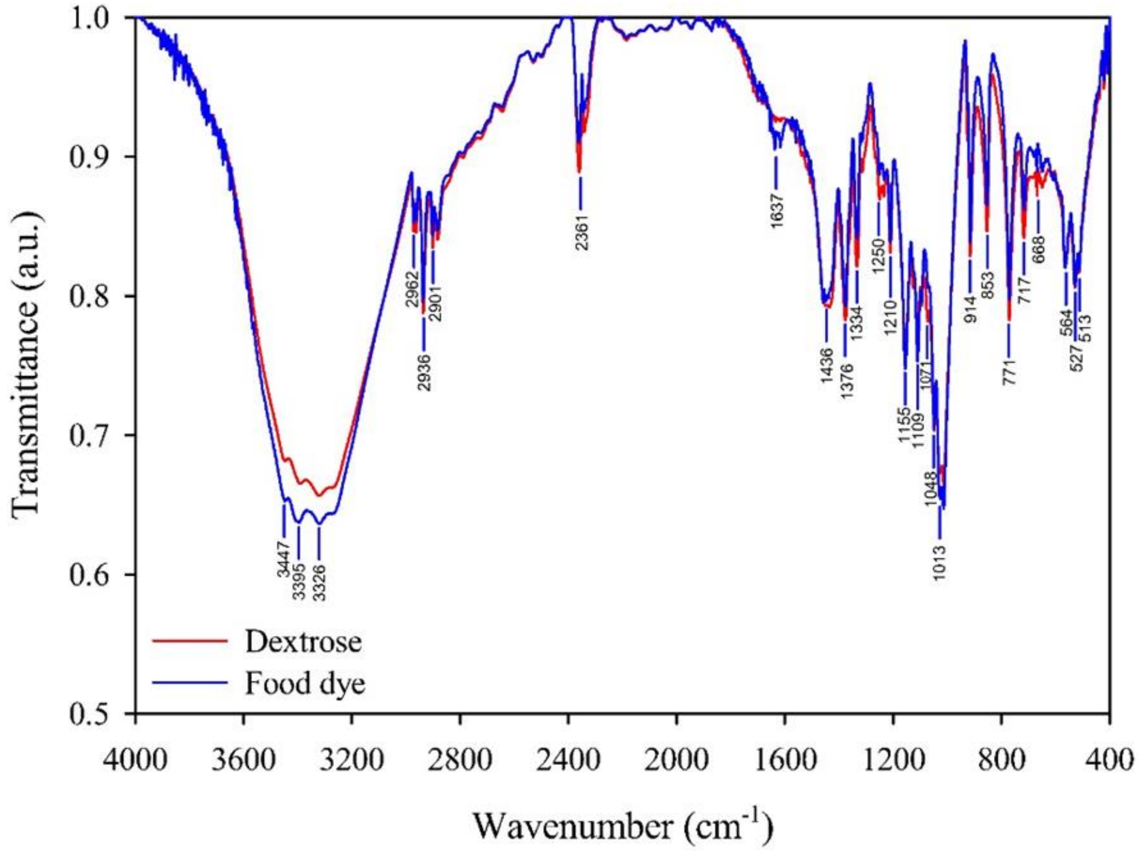

Figure S2. a) The ATR FTIR spectra of the food dye and of D-glucose (dextrose)

Figure S2. b) The KBr-pellet IR spectrum of food dye and D-glucose

The high amount of dextrose in food dye implicates the overlapping spectra of dextrose and food dye that may be observed from Figure S2 a) and b). Due to the overlapping spectra of dextrose and food dye, there is no specific peak indicating the confirmation of indigo carmine that may be observed. Thereby, only a small amount of indigo carmine is shown to be in this food dye which is composed of indigo carmine and dextrose. For this reason, we find that indigo carmine cannot be detected by such bulk sample techniques as ATR and KBr.

The following table lays out the assignation of dextrose peaks in both the FTIR spectra.

Table S1: The assignation of the dextrose peaks of the infrared spectrum

			D-glucose[S1]		
IR	α-D	β-D-M	Assignment	ATR	KBr
3410	3418	3146	ν OH	3248	3326,3347,3395
3393	3408	3123	ν OH	2970	2962
2944	2963	3099	ν_s CH of C_2	2937	2936
2913	2939	3085	ν_{as} CH of C_1	2902	2901
1460	1441	1465	δCH_2+δOHC+δCCH	1427	1436
1382	1352	1390	δOCHC+δCOH+δCCH	1374	1376
1340	1333	1320	δCCH+δOHC	1333	1334
1224	1218	1278	δCH+δOH in plane	1249	1250
1203	1220	1232	δCH+δOH in plane	1209	1210
1149	1137	1205	ν CO + ν CC	1155	1155
1111	1116	1162	ν CO	1109	1109
1050	1058	1088	ν CO + ν CC	1072	1071
1025	1026	1066	ν CO	1047	1048
995	944	1032	ν CO + ν CC	1014	1013
915	900	993	ν CO + ν CC+νas ring of pyranose	915	914
837	834	909	δCH	852	853
776	762	815	δCCO+δCCH	771	771
622	619	694	CH_2	717	717

KBr, a more sensitive technique compared to ATR, yielded almost entirely similar peak positions in the IR spectra. Only the bands corresponding to stretching OH vibrations are not in the same positions, as well as for the vibrations of δCH_2+δOHC+δCCH [S1]. The peak positioned at 564 cm^{-1} is of CCO , OCO and CCH, while the peak positioned at 527 cm^{-1} is of τCO, OCO, CO, τCCO vibrations. The peak positioned at 513 cm^{-1} is of τCO and

$C_4C_5O_5$[S2] . The peak that appears at 1637 cm^{-1} is absorbed water in the material. The peaks positioned at 2343 cm^{-1}, 2361 cm^{-1} and 668 cm^{-1} belong to the uncorrected CO_2.

RAMAN SPECTROSCOPY

Table S2. The l engths and angles between the atoms in the indigo carmine molecule

Bound	Bound length/Å	Bond	Angle/ °	Bond	Angle/ °
O_{27}=S_{25}	1.49635	O_{27}=S_{25}=O_{26}	111.06899	H_{30}-C_{10}-C_7	120.77773
S_{25}=O_{26}	1.49873	O_{26}=S_{25}=O_{28}	110.21532	C_{10}-C_7-C_6	120.67618
S_{25}=O_{28}	1.49802	O_{27}=S_{25}=O_{28}	111.10462	C_{10}-C_{11}-S_{14}	118.57470
S_{25}-C_{22}	1.79833	O_{25}-C_{22}-C_{21}	118.57478	C_{12}-C_{11}-S_{14}	119.89087
C_{22}-C_{21}	1.39590	C_{22}-C_{21}-H_{34}	121.40348	C_{11}-S_{14}=O_{15}	108.03586
C_{21}-H_{34}	1.08608	H_{34}-C_{21}-C_{20}	120.77773	O_{15}=S_{14}=O_{17}	110.21540
C_{21}-C_{20}	1.39306	C_{21}-C_{20}-C_{19}	120.67618	O_{17}=S_{14}=O_{16}	111.07746
C_{20}-C_{19}	1.41859	C_{20}-C_{19}-C_{24}	121.36016	O_{15}=S_{14}=O_{16}	111.09590
C_{19}-C_{24}	1.39516	C_{19}-C_{24}-H_{36}	121.57856	O_{26}=S_{25}-C_{22}	108.00656
C_{24}-H_{36}	1.08559	H_{36}-C_{24}-C_{23}	120.82717	O_{27}=S_{25}-C_{22}	108.25944
C_{23}-C_{24}	1.39787	C_{24}-C_{23}-H_{35}	120.45778	C_{22}-C_{21}-C_{20}	117.81876
C_{23}-H_{35}	1.08497	H_{35}-C_{23}-C_{22}	118.52595	C_{19}-C_{24}-C_{23}	117.59427
C_{23}-C_{22}	1.40501	C_{23}-C_{22}-C_{21}	121.53435	C_{24}-C_{23}-C_{22}	121.01627
C_{20}-C_2	1.47023	C_{20}-C_2=O_1	130.53313	C_{20}-C_2-C_3	104.04648
C_2=O_1	1.23341	O_1-C_2-C_3	125.42037	C_{19}-C_{20}-C_2	107.69036
C_2-C_3	1.49639	C_2-C_3-N_{18}	108.40419	C_6-N_5-C_4	110.27459
C_3-N_{18}	1.37710	C_3-N_{18}-H_{33}	122.17508	N_5-C_4-C_8	108.40419
N_{18}-H_{33}	1.01219	C_3-N_{18}-C_{19}	110.27459	C_{11}-S_{14}=O_{16}	108.25949
N_{18}-C_{19}	1.38540	N_{18}-C_{19}-C_{20}	109.58438	S_{25}-C_{22}=C_{23}	119.09087
C_3=C_4	1.36648	C_2-C_3=C_4	126.37752	C_{21}-C_{20}-C_2	131.63345
C_4-N_5	1.37710	N_{18}-C_3=C_4	125.21829	C_{24}-C_{19}-N_{18}	129.05546
N_5-H_{29}	1.01134	C_3=C_4-N_5	125.21829	C_{13}-C_6-C_7	121.36016
N_5-C_6	1.38540	C_3=C_4-C_8	126.37752	C_{22}-S_{25}-O_{26}	108.00656
C_6-C_7	1.41859	C_4-C_8=O_9	125.42038	C_{22}-S_{25}-O_{27}	108.25944
C_7-C_8	1.47023	C_4-C_8-C_7	104.04648	C_{22}-S_{25}-O_{28}	108.06455
C_8-O_9	1.23341	O_9=C_8-C_7	130.53314	C_{11}-S_{14}-O_{16}	108.25944
C_8-C_4	1.49639	C_8-C_7-C_6	107.69036	C_{11}-S_{14}-O_{15}	108.03586
C_6-C_{13}	1.39516	C_7-C_6-N_5	109.58438	C_{11}-S_{14}-O_{17}	108.03539

C_{13}-H_{32}	1.08559	C_6-N_5-H_{29}	127.58039
C_{13}-C_{12}	1.39787	H_{29}-N_5-C_4	122.14502
C_{12}-H_{31}	1.08497	N_5-C_6-C_{13}	129.05546
C_{12}-H_{11}	1.40501	C_6-C_{13}-H_{32}	121.57856
C_{11}-C_9	1.39590	H_{32}-C_{13}-C_{12}	120.82717
C_{10}-H_{30}	1.08608	C_6-C_{13}-C_{12}	117.59427
C_{10}-C_7	1.39306	C_{13}-C_{12}-H_{31}	120.45778
C_7-C_6	1.41859	H_{31}-C_{12}-C_{11}	118.52595
C_{11}-S_{14}	1.79833	C_{13}-C_{12}-C_{11}	121.01627
S_{14}=O_{16}	1.49634	C_{12}-C_{11}-C_{10}	121.53435
S_{14}=O_{17}	1.49817	C_{11}-C_{10}-H_{30}	121.40348
S_{14}=O_{15}	1.49856	C_{11}-C_{10}-C_7	117.81876

Table S3. The theoretical and experimental values of the vibration bands obtained in this current research compared to the literature [25 from manuscript]

This research		Literature[25]				
	B97D		B3PW91		B3LYP	
Exp.	cc-pVTZ	Exp.	LANL2DZ	6-311++G**	LANL2DZ	6-311++G**
1240	1235	1240	1239	1243	1265	1244
1296	1293	1290	1288	1280	1288	1296
1346	1348	1344	1357	1345	1343	1352
1576	1588	1576	1511	1509	1621	1616
1626	1625	1623	1640	1632	1635	1634
1697	1703	1698	1687	1701	1669	1681

Video1.mp4 to video4.mp4 record the molecule's vibration for the bands as indicated by the arrows in Figure S3.

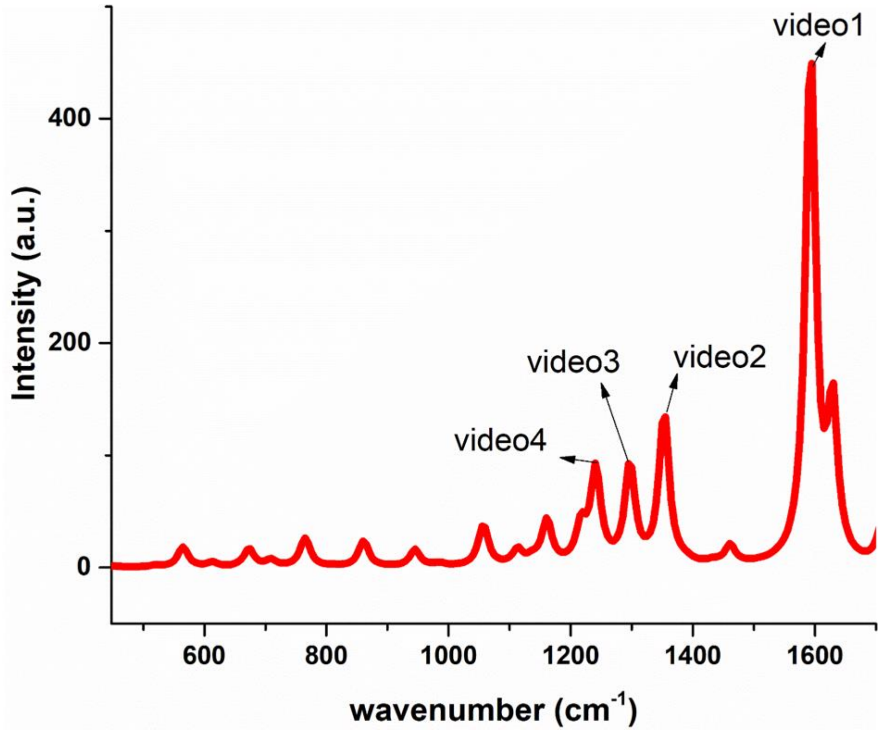

Figure S3. The multimedia (video1.mp4 to video4.mp4) of complex vibrations of the indigo carmine functional groups obtained for the most intensive peaks

The Gaussian output log file (4447431_opt_raman_Na.log) may be used to read the Raman vibrations of indigo carmine in Facio software.

UV/VIS METHOD

According to the UV/Vis spectra obtained for an aqueous solutions of indigo carmine, an analytical curve was plotted, as presented in Figure S4. The absorbance of the measured aqueous solution of the mixture of indigo carmine and dextrose has been marked in the UV Vis analytical curve. The points chosen to create the analytical curve are marked in orange.

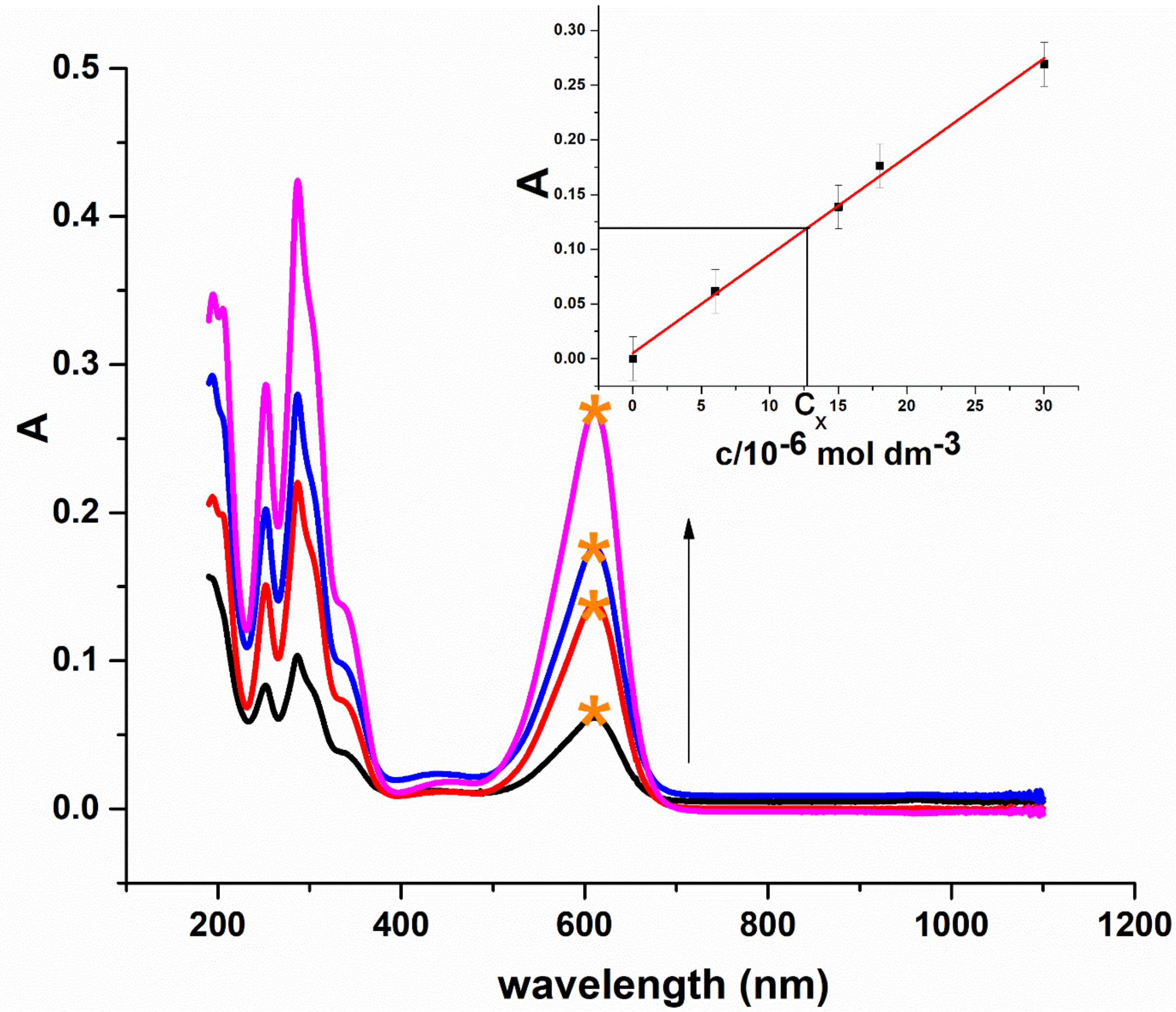

0.5
0.4
0.3
0.2
0.1
0.0
A
200
400
600
800
1000
1200
wavelength (nm)
0.30
0.25
0.20
0.15
0.10
0.05
0.00
A
0
5
10
Cx
15
20
25
30
c/10-6 mol dm-3

Figure S4. The indigo carmine standard concentrations' UV/Vis spectrum of 3.00 × 10^{-5} mol dm^{-3}; 1.80 × 10^{-5} mol dm^{-3}; 1.50 × 10^{-5} mol dm^{-3} and 0.60 × 10^{-5} mol dm^{-3} (the arrow indicating the rise of concentration). Insert: UV/Vis calibration curve (the dependence of A at λ_{max} = 611 nm as a function of the concentrations).

Although t he D-glucose yields no |UV/Vis spectrum, the indigo carmine does , as presented in Figure S5.

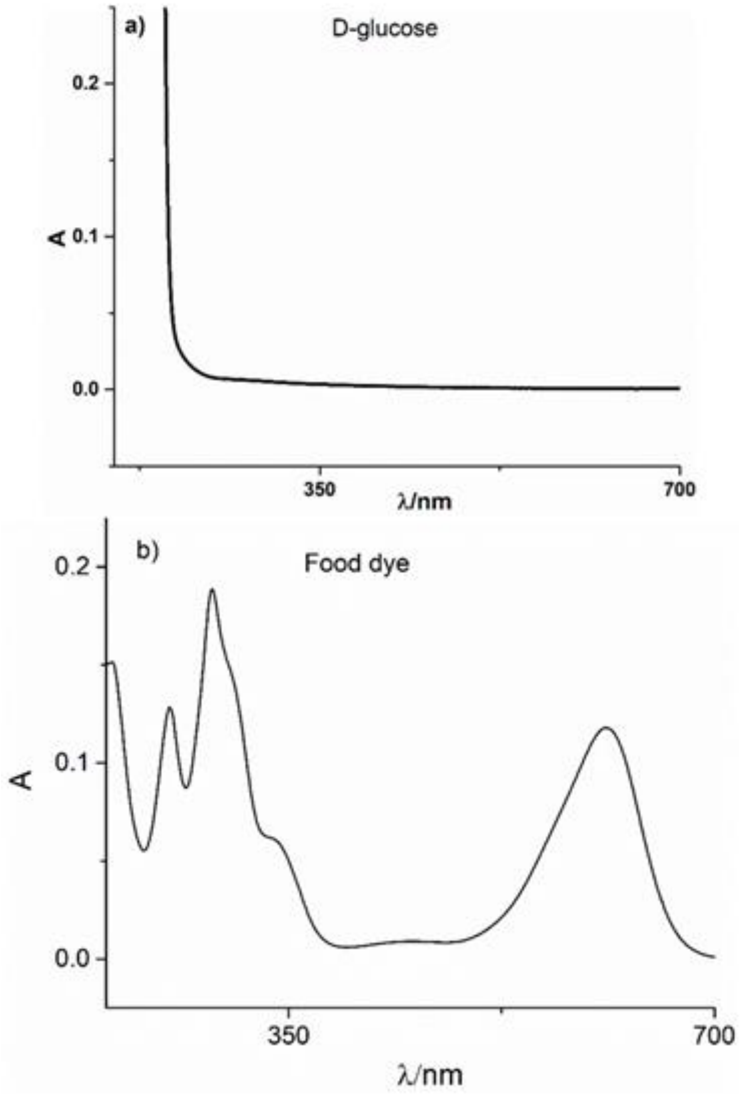

Figure S5. The UV/VIS spectrum of D-glucose **a)** food dye (mixture of indigo carmine and D-glucose) **b).**

THE BRIGGS-RAUSCHER METHOD

The recorded Pt-potential vs. time series (or oscillogram) of the BR reaction free of analyte is shown at **Figure S6 a) in three oscillograms**. The influence of D-glucose (added at 30 s from the beginning of the BR reaction) to the oscillating BR reaction, as well as the basic BR oscillogram, are presented in **Figure S6 b)**.

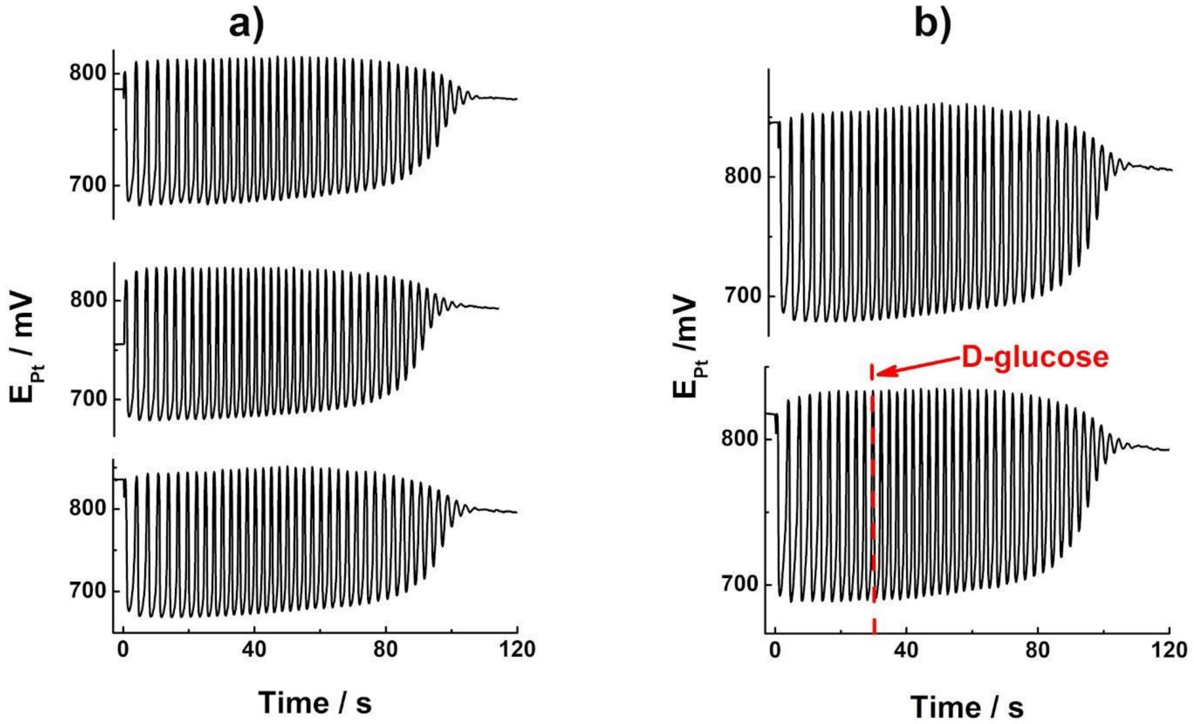

Figure S6. The Basic BR oscillogram in three oscillograms a) and the influence of D-glucose (100 µl, 0.1 mol/dm^3) to BR oscillogram b). The initial reactant concentrations in the system were $[CH_2(COOH)_2]_0$ = 0.0789 mol dm^{-3}, $[MnSO_4]_0$ = 0.00752 mol dm^{-3}, $[HClO_4]_0$ = 0.03 mol dm^{-3}, $[KIO_3]_0$ = 0.0752 mol dm^{-3} and $[H_2O_2]_0$ = 1.176 mol dm^{-3}.

S REFERENCES:

[S1] Analysis of the structure and vibrational spectra of glucose and fructose M.Ibrahim, M. Alaam, H. El-Haes, A. F. Jalbout, A. de Leon, Analysis of the structure and vibrational spectra of glucose and fructose, Eclet. Quim. 31(3) (2006) 15-21.

https://doi.org/10.1590/S0100-46702006000300002

[S2] M.V.Korolevich, R.G.Zhbankov, V.V.Sivchik, Calculation of absorption band frequencies and intensities in the IR spectrum of α-d-glucose in a cluster, J. Mol. Struct., 220 (1990) 301-313. https://doi.org/10.1016/0022-2860(90)80120-9
